# IDCube Lite: Free Interactive Discovery Cube software for multi- and hyperspectral applications

**DOI:** 10.1255/jsi.2021.a1

**Published:** 2021-05-05

**Authors:** Deependra Mishra, Helena Hurbon, John Wang, Steven T. Wang, Tommy Du, Qian Wu, David Kim, Shiva Basir, Qian Cao, Hairong Zhang, Kathleen Xu, Andy Yu, Yifan Zhang, Yunshen Huang, Roman Garnett, Maria Gerasimchuk-Djordjevic, Mikhail Y. Berezin

**Affiliations:** a Department of Radiology, Washington University School of Medicine, 4515 McKinley Ave, St Louis, MO 63110, USA; b Department of Computer Science and Engineering, Washington University, 1 Brookings Hall, St Louis, MO 63110, USA; c Art and Design Department, Missouri State University, 901 S. National Ave, Springfield, MO 65897, USA; d HSpeQ LLC, 4340 Duncan Ave, St Louis, MO 63110, USA

**Keywords:** hyperspectral, multispectral, spectral imaging, IDCube, segmentation, geospatial, biomedical

## Abstract

Multi- and hyperspectral imaging modalities encompass a growing number of spectral techniques that find many applications in geospatial, biomedical, machine vision and other fields. The rapidly increasing number of applications requires convenient easy-to-navigate software that can be used by new and experienced users to analyse data, and develop, apply and deploy novel algorithms. Herein, we present our platform, IDCube Lite, an Interactive Discovery Cube that performs essential operations in hyperspectral data analysis to realise the full potential of spectral imaging. The strength of the software lies in its interactive features that enable the users to optimise parameters and obtain visual input for the user in a way not previously accessible with other software packages. The entire software can be operated without any prior programming skills allowing interactive sessions of raw and processed data. IDCube Lite, a free version of the software described in the paper, has many benefits compared to existing packages and offers structural flexibility to discover new, hidden features that allow users to integrate novel computational methods.

## Introduction

Multi- and hyperspectral imaging (HSI) modalities have emerged as an exciting opportunity to explore optical properties of objects and discover hidden features not accessible by other techniques. In contrast to the traditional spatial images produced by conventional cameras, spectral imaging generates 3D datasets (datacubes), with spatial and spectral dimensions. With each pixel containing information on the entire medium or high-resolution spectrum, spectral imaging provides abundant information about individual chromophores and their interactions that contribute to the location, intensity and alteration of the optical signal, significantly better than monochromatic or traditional colour cameras.^1,2^ This spectral imaging approach leads to a vastly improved ability to classify and differentiate the objects based on their spectral features, enabling even small, otherwise unnoticeable, features to be amplified.

In the last decade, spectral imaging has expanded from a narrow niche accessible only to a handful of organisations in academic and governmental research facilities to a broad range of commercial and clinical institutions. As spectral imaging hardware (benchtop scanners, handheld HSI cameras, drones etc.) have become more available,^3–5^ the number of spectral imaging studies has increased tremendously,^6–11^ reaching more than 25,000 publications in 2019.

Meaningful analysis of datacubes is the most critical and time-consuming step in many current applications. The high dimensionality of spectral imaging data and their large data sizes (often >1 GB) gives an excellent opportunity to learn more about the subject; however, the extensive analyses (i.e. pretreatments, identifying appropriate algorithms etc.) of these datasets present the strongest barrier to the imaging workflow. Despite the progress in computational speed and algorithm development, efficient computational approaches suitable for general use applications are lacking. Well-known software packages, such as ENVI,^12^ are comprehensive; however, they are mostly used for remote sensing applications. A handful of free hyperspectral software packages with strong innovative algorithms (e.g., Gerbil^13^ or MultiSpec^14^) are more suitable for experts and are limited to geospatial applications. Introduced in 2020, the Hyperspectral Toolbox from MATLAB^15^ has very few algorithms, is limited in the number of supported file formats and is largely command-line based. Other packages analysing spectral imaging data are attached to specific hardware to visualise the acquired data and usually do not provide the desired level of data mining and processing capability. As such, there is a tangible need for a more universal, powerful computational platform that enables comprehensive and rapid data mining for a variety of platforms in order to offer real-time efficiency and throughput for the majority of applications.

Herein, we present IDCube, Interactive Discovery Cube, Lite (https://www.idcubes.com), a highly versatile software that performs a large number of essential operations in the spectral imaging domain and enables image analysis for users across a range of technical proficiencies. The goal of the software is to make spectral imaging accessible to new and current users that focus on obtaining useful results rather than (but not excluding) developing algorithms. The strength of the proposed software lies in its intuitive design that enables the user to perform high-level data analysis as well as develop their own algorithms via a visual, interactive interface. Built around a collection of spectral imaging algorithms, the software facilitates the search of hidden information inside large datasets providing a new experience of data analysis. The entire program can be operated without prior programming skills and allows almost any currently used data formats to be processed.

**Figure 1.**
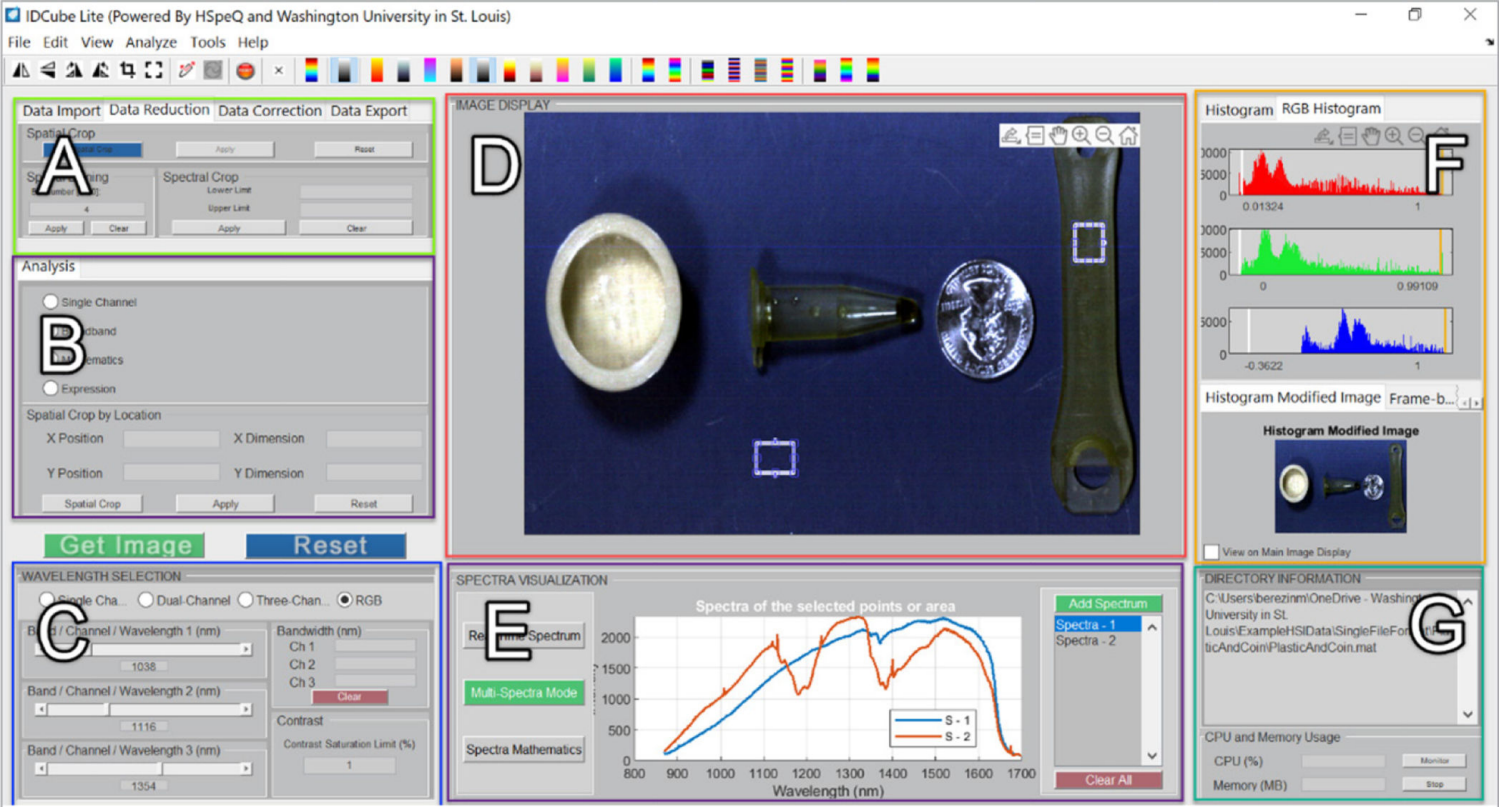
Front end of the IDCube Lite hyperspectral software. A: import/export, data reduction, data correction; B: image algebra selection with preselected and user-developed functions; C: wavelength and bandwidth selection, image optimisation; D: image visualiser, E: spectral analysis of selected ROIs; F: image enhancement via histogram manipulation; G: information about the image, the consumed computational resources for the performed tasks.

The overall workflow of IDCube Lite is shown in Table 1. The processing steps are centred around the visualisation module that presents the original and processed data in a 2D format. The front end of the software is shown in Figure 1.

The core of the software is written in MATLAB with an extensive number of built-in image processing functions. The compiled software can be run on any computer with a Windows or Mac Operating System, without having a MATLAB license. An entirely free version of the software IDCube Lite is available for download from a secure cloud location, www.idcubes.com. An extensive library of video tutorials is available from the learning module of the software website. Below we will focus on the technical capabilities of IDCube Lite and demonstrate its performance through several experiments in the geospatial, machine vision and biomedical fields. A brief description of the datasets used in this paper is given in Table 2.

## Features

IDCube Lite enables visual analysis of many datasets from a variety of formats. The platform has been constantly improving since its launch in September 2020 and now includes more than 50 integrated algorithms for data processing and data visualisation. These algorithms are grouped into the following categories: Input/Output, Data Reduction, Image Visualisation, Spectral Analysis, Principal Component Analysis (PCA)/Maximum Noise Fraction (MNF), Segmentation and Spectral Matching. The selected algorithms are optimised for processing time and do not exceed several seconds when processing a 1 GB file on a standard desktop computer. The descriptions of the major features are given below.

### Input/output module

IDCube Lite supports a wide range of image formats, including Raw/HDR, DAT, TIFF, JPEG2k, PNG and several others. The list of the available formats is given in Table 3. The imported files (datafile+header file) are first converted, then saved in the same directory as a single MATLAB-type file. The single file that combines raw data and the channel assignment information (wavelength vector) automatically opens in the IDCube Lite interface after the conversion is complete. IDCube Lite supports imaging data from a variety of bench-type HSI systems that utilise a Raw/HDR format as well as from satellites offering data in JP2 and TIFF. In addition to HSI datasets, the software can also treat standard RGB images and thus presents an interesting opportunity to apply sophisticated computational HSI techniques to common pictures.

The software can open up to ten hyperspectral datasets simultaneously for all datasets with the same dimensions. If the images have different spatial dimensions, they can be first cropped using the spatial cropping function. Analysing multiple datasets in one setting enables data comparison and the same processing for data from longitudinal studies.

### Preprocessing module

The preprocessing module includes the ability to perform binning in the spatial domain to decrease the size of the file and reduce noise. The module offers interactivity through spatial cropping, spectral cropping and flipping/transposition/rotation. All preprocessing functions are applied globally to the entire dataset. The DATA CORRECTION tool removes undesirable artefacts caused by scattering of light that produces undesirable artefacts. The correction algorithms include multiplicative scattering correction (MSC)^16^ and standard normal variate (SNV)^17^ functions. This feature is especially useful for biomedical imaging; for example, to decrease scattering artefacts from skin. Other preprocessing steps to decrease the size of the image file via removal of highly correlating spectral bands and extracting endmember signatures from hyperspectral data^18^ will be implemented in future versions.

### Visualisation module

This module is central to image processing and presents three-dimensional (3D) datasets through a set of two-dimensional (2D) images. The 2D images are generated through the three wavelength channels. The wavelength selection can also be appended with a preselected bandwidth rather than the default bandwidth of 1. The produced monochromatic image can be colour optimised by applying an appropriate lookup table (LUT) from more than 20 available LUTs. The visualisation is further adjusted via the histogram tool. The BROADBAND function enables visualising the image within a specific wavelength range. When this function is selected, the wavelengths w1 and w2 will provide the boundaries for the spectral range (i.e. the image produced by selection w1 = 950 nm, and w2 = 1400 nm would correspond to an image acquired by the camera over the 950–1400 nm range). The MATHEMATICS mode in conjunction with the TWO-CHANNEL modes enables the user to conduct image algebra. Preset functions include division of one wavelength channel over another, subtraction, logical functions etc. The EXPRESSION module allows the user to type a custom mathematical function or select from the list of implemented functions specified in the Mathematics Sheet for Expressions available from the software website (https://www.idcubes.com/tutorials). The SINGLE CHANNEL, TWO-CHANNEL and THREE-CHANNEL tools allow users to scroll the images through the individual wavelengths. The RGB mode enables the user to combine up to three wavelengths into a pseudo-RGB image. Each wavelength can be used with the specific bandwidth. The HISTOGRAM optimisation and the CONTRAST ADJUSTMENT fields enable the user to improve the image contrast. One of the unique features of IDCube Lite is to present the dataset as a movie where each frame represents an image at a specific wavelength or with a formula applied. This feature implemented in the FRAME BY FRAME display function radically minimises the amount of user interaction and prevents image processing fatigue. The image with the entire dataset can be flipped, transposed and rotated. The produced 2D image can be also copied, zoomed, panned and saved.

### Spectral analysis module

The spectral analysis module enables the user to visualise and process spectral information from individual pixels and interactively select regions of interests (ROIs). In the REAL-TIME spectral mode, the module enables visualisation of spectra from individual pixels by moving the cursor over the image. The module automatically recognises a spectral range and scales the dimensions of the spectral plot. In the MULTI-SPECTRA mode, the user is prompted to select one or more ROIs. The spectrum for each ROI reflects the average spectrum across the selected area. SPECTRA MATHEMATICS enables the user to perform basic math functions, i.e. subtraction and division of the spectra, spectra normalisation and calculations of the first and second derivatives. The user can compare the spectral output from the selected ROIs using spectral correlation, spectral information divergence^19^ or spectral angle^20^ functions. Relatively high spectral correlation values, low divergence and low spectral angles suggest regions with similar spectral properties indicating the objects belong to the same class of objects with similar optical profiles or same materials. All spectra can be zoomed, panned and exported to Excel or other data analysis software.

### Principal Component Analysis (PCA) module

**Figure 2.**
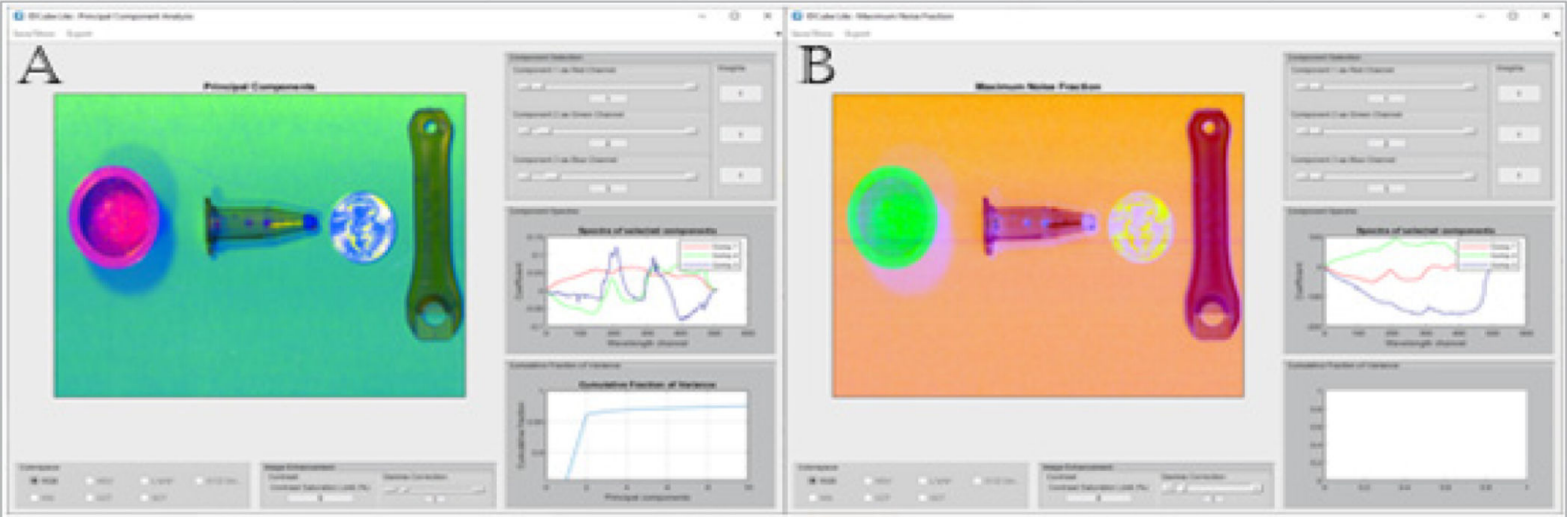
Preprocessing tools. A. Principal Component Analysis of a hyperspectral dataset. First, second and third principal components are combined in a pseudo-colour RGB image. The module also presents the spectra of selected components and a cumulative fraction of variance. The image can be improved by adjusting the contrast and gamma correction. B. Maximum Noise Fraction of the same data shows the partial removal of the shadow. Example file can be downloaded through IDCube Lite under File/Example iles/Plastic And Coin.

The PCA module (Figure 2) computes associations between data points and converts a dataset of potentially correlated variables into a new set of linearly uncorrelated principal components.^21,22^ Up to three principal components can be selected by the user to generate a pseudo-colour RGB image, where the selected components are assigned to three colours, red, green and blue. Objects with the same colour indicate high similarity between two subjects. For example, a centrifuge tube and a wrench shown in Figure 2 are apparently made from the same material, since their PCA-based pseudo-colours are almost identical. The pseudo-colour RGB image can be further adjusted through changing the WEIGHT of the individual component, adjusting the CONTRAST to the whole image and applying the GAMMA CORRECTION. The gamma correction^23^ helps to improve the contrast if the image is too dark or too bright. The value of the gamma correction can take any value between 0 and infinity (up to 10 using a slider, or to any value if typed). If the gamma is less than 1, the output image is brightened, for gamma greater than 1, the output image is darkened.

In general, the total number of principal components is equal to the number of wavelength channels. Since most of the information is in the first few components, the IDCube Lite version limits the number of stored principal components to 20. The COMPONENT SPECTRA window enables the user to examine the eigenvectors visually to check if relevant features may be extracted. The module also presents the cumulative fraction of variance. In the given example, the first two components carry >96 % of the variance. PCA transforms the original datacube into a new datacube with the same spatial dimensions and changes the Z-axis from wavelengths to principal component scores. In that case, PCA can be also considered as a function that decreases the size of the data, since only very few principal components (first 20, for example) can be used. The new, smaller datacube can be exported back to the VISUALISATION panel and analysed using implemented algorithms.

A variation of the PCA method is an MNF.^24^ The MNF transform has advantages over the PCA transform because it takes the noise information in the spatial domain into consideration. For example, the shadows seen in Figure 2A can be removed to some extent using the MNF function implemented in IDCube Lite as shown in Figure 2B.

### Segmentation module

**Figure 3.**
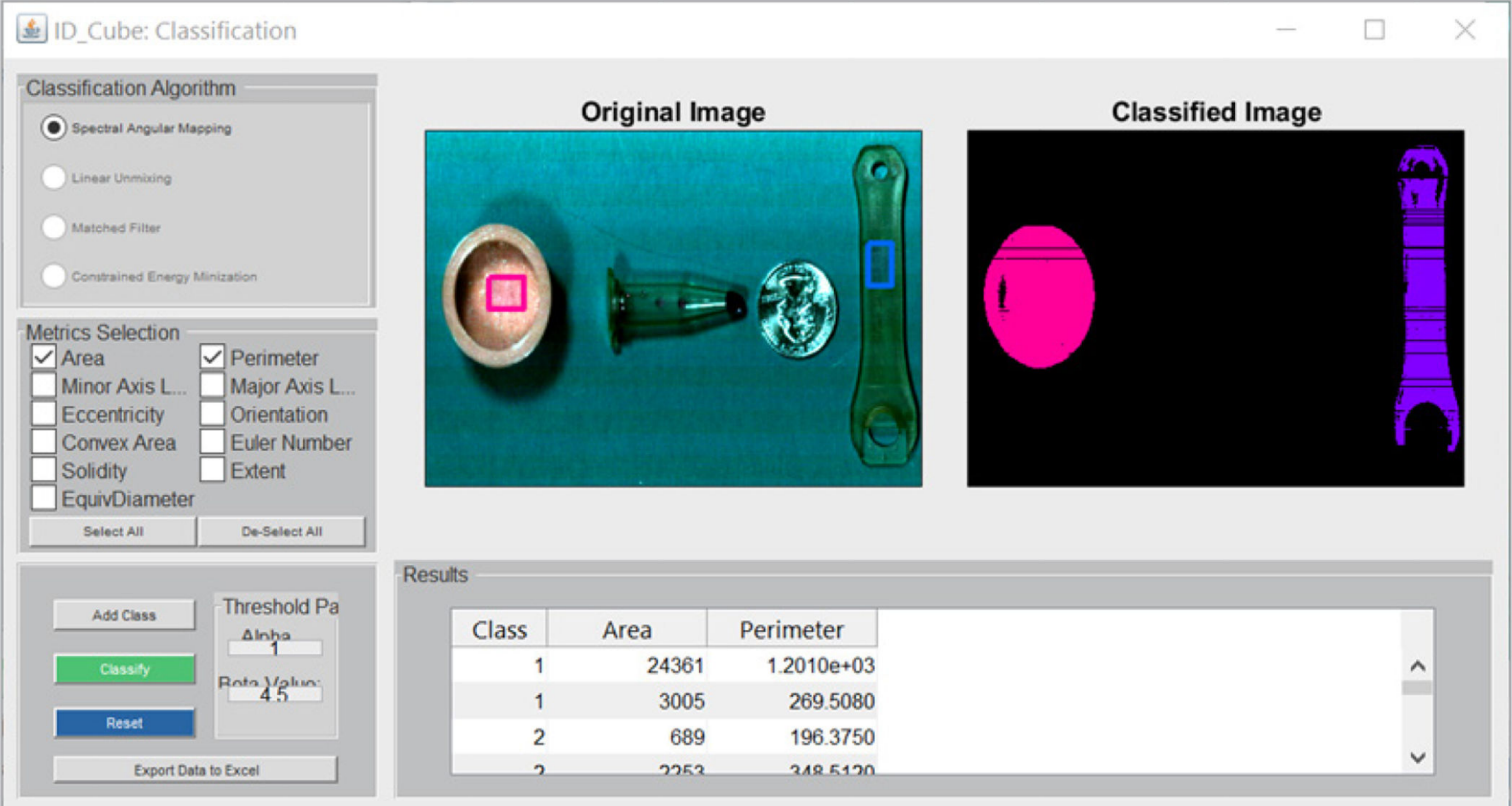
Image Classification Module. In the Spectral Angular Mapping, the user interactively selects different areas (classes) from the 2D image. Although there is no limit to the number of classes, the module works best for one or two classes. The dark lines correspond to “bad pixels” that are often seen in the SWIR cameras. This may be due to a damaged pixel in the sensor array.

The segmentation module in IDCube Lite (Figure 3) enables minimally supervised classification of a dataset from any of the preprocessing algorithms (spatial, spectral cropping, binning, PCA etc.). In a typical workflow, the user selects one of the classification algorithms [i.e. based on the improved Spectral Angular Mapper (SAM) currently implemented in IDCube Lite] and the metrics (i.e. area, perimeter) then draws an area (class) on the image passed from the Visualisation module. Areas with similar spectral properties (that have low values of spectral angle) are represented by the same colour and quantified according to the selected metrics. The example shown in Figure 3 illustrates this method for classification. One can notice that the method is highly sensitive to even small spectral changes, where the vial and the wrench can be separated even though their spectral correlation value is 0.99 (as measured using the SPECTRAL ANALYSIS module, see above).

### Speed and resources

**Figure 4.**
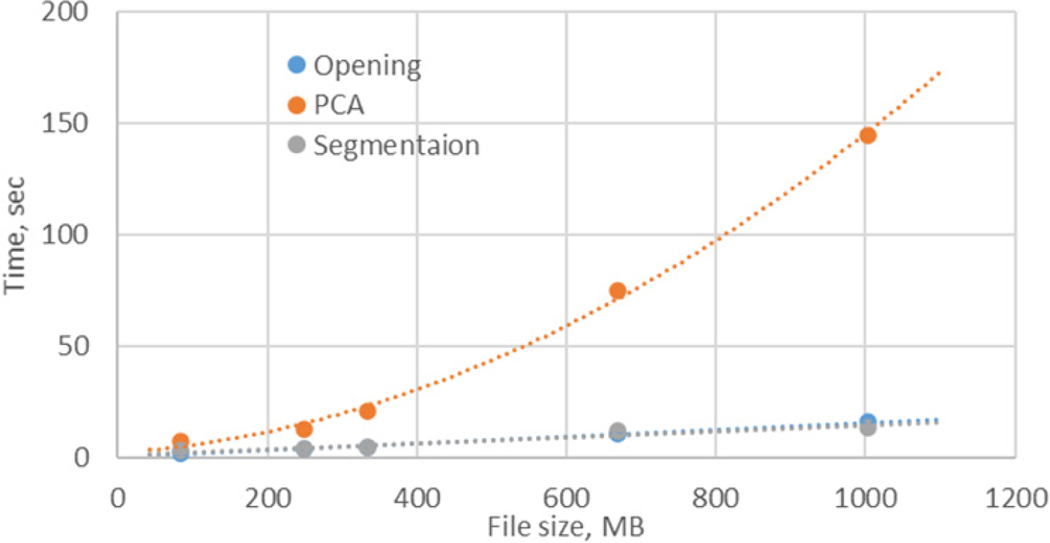
Time for file opening, PCA processing and segmentation with SAM with two classes. PC: Dell Vostro 5090, Intel Core i7–9700CPU, 3 GHz, RAM 24 GB, Windows 10.

Due to the large size of hyperspectral datasets, many of the functions of the software are optimised for working with large datasets exceeding several hundred megabytes. Figure 4 presents the speed of the most demanding functions in typical hyperspectral dataset analysis. A file of about 1 GB can be opened in less than 10 s on a standard home PC and significantly faster on more powerful computers. Most of the visualisation and spectral analysis functions are performed almost instantaneously. PCA is the most time-consuming with the processing time significantly and non-linearly increasing with the size of the file, reaching more than 2 min for a 1-GB file in our test PC (Dell Inc., Vostro DT 5090, Intel Core i7–9700, 8 Core, GB (1 × 8 GB) DDR4 2666 MHz UDIMM).

## Examples

All examples mentioned in this paper (Table 2) and other datasets can be downloaded from IDCube Lite directly (File – Download examples) or from the website https://www.idcubes.com/examples.

### Biomedical applications

With the development of clinically relevant hyperspectral imaging instrumentation, HSI has emerged as a powerful tool for investigating complex biological systems.^1,25^ Biological tissues in the visible range often do not provide sufficient contrast to distinguish the structures of interest and, therefore, require contrast agents for contrast enhancement. Hyperspectral imaging, with its inherently higher sensitivity to minor changes, can replace some of the elaborate staining techniques and significantly accelerate the pathological practice both *in vitro* and *in vivo*. Clinical examples include histopathology,^26^ dermatology,^27^ ophthalmology,^28^ gastroenterology,^29^ oncology^30,31^ and deep tissue imaging with hyperspectral shortwave infrared (SWIR, 900–2200 nm) due to a high penetration of SWIR photons through the skin and the tissue.^32^

**Figure 5.**
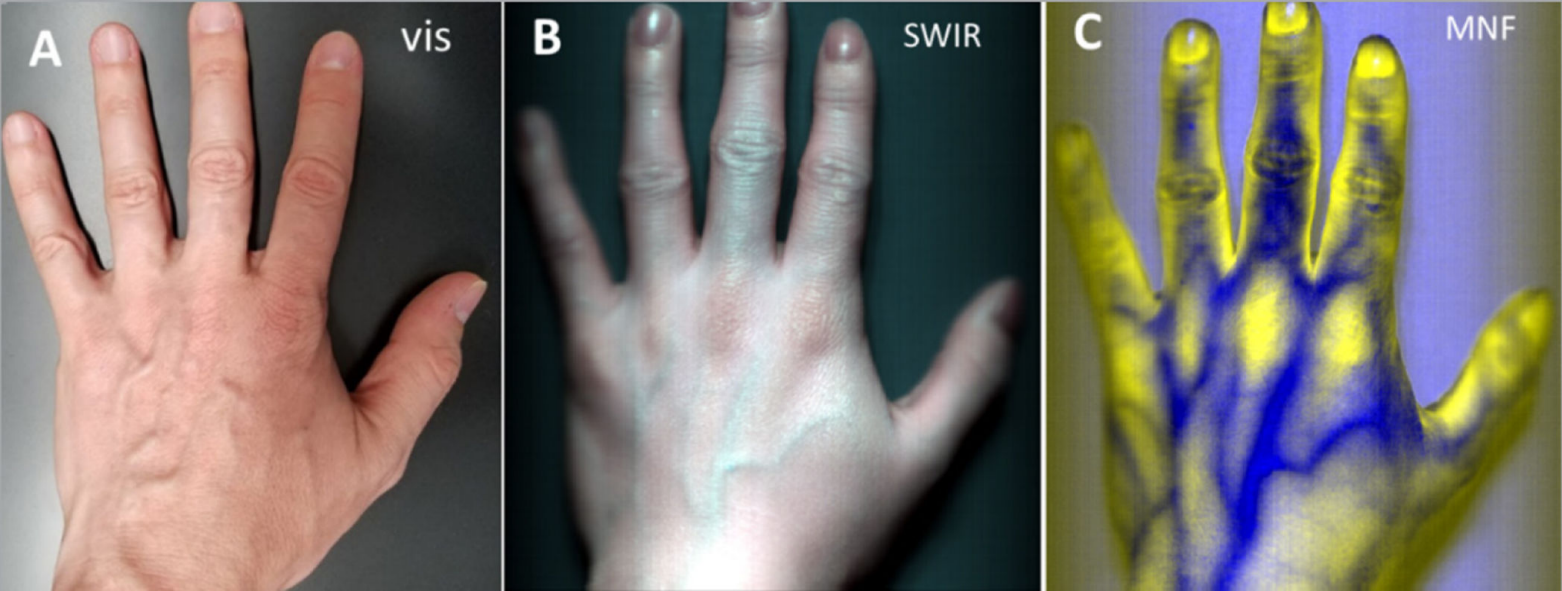
Hyperspectral imaging of a hand. A: made by a conventional visible camera; B: using a hyperspectral SWIR imager. Pseudo-RGB image at 1070 nm (red), 1260 nm (green), 1320 nm (blue); C: MNF function applied to the HSI dataset. Pseudo-RGB image at #3 (red), #3 (green), #2 (blue) components. Example file can be downloaded through IDCube Lite under File/Example Files/Hand SWIR.

The example shown in Figure 5 illustrates the utility of IDCube Lite to better visualise blood vessels. First, the acquired dataset was spectrally cropped to eliminate the noisy wavelength channels. Since our imaging system based on the InGaAs detector (Ninox 640, Raptor Photonics) in combination with the spectrograph N17E (Specim Inc.) used in this study typically has lower sensitivity below 900 nm and above 1700 nm, these wavelengths were excluded. The RGB mode was then selected, and the wavelengths were manually adjusted to visualise the blood vessels. The image made with a conventional colour camera does not show the blood vessels (Figure 5A). The visualisation of the resulting image was further improved by adjusting corresponding colour band histograms. The resulting image shown in Figure 5B presents blood vessels in a greater contrast than the visible image. The dataset was then treated with the MNF function selected from the ANALYSE tab. Similar to the PCA module described above, the software enables the user to select individual components and presents them in the pseudo-RGB format (Figure 5C). High contrast for blood vessels was achieved by using components #2 and #3.

In addition to HSI, IDCube can handle other spectral imaging modalities commonly used in preclinical and clinical studies, such as Raman and Fourier transform infrared spectroscopies, and fluorescence-lifetime imaging microscopy.^33^

### Environmental applications

**Figure 6.**
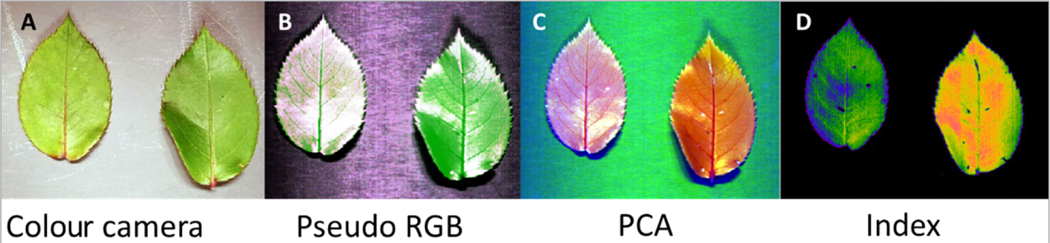
Hyperspectral imaging of leaves with IDCube Lite. A: image of two similar leaves obtained with a conventional colour camera; the leaf on the left came from the plant exposed to a drying condition; B: contrast improvement with a three-band approach in a pseudo-RGB image: 1421 nm (red), 1351 nm (blue) and 1476 nm (green); C: PCA with three components #1 (red), #2 (green), #3 (blue) in a pseudo-RGB image; D: monochromatic image reflecting a drought index (1529–1416 nm)/(1519 nm + 1416 nm). Example file can be downloaded through IDCube Lite under File/Example Files/Rose Leaves.

HSI of plants provides solutions to a large number of challenges from identifying environmental issues to monitoring crops yield and diseases^34^ and even detection of contaminations^35^ and minerals. The optical signature of plants and especially leaves is an important monitoring and predictive parameter for a variety of biotic and abiotic stresses. Figure 6 illustrates an application of IDCube Lite on a dataset from leaves with different moisture levels. The right leaf on each image belongs to a plant grown under normal conditions, and the left leaf was exposed to a drying element. This treatment was used to mimic a drought condition in order to showcase the effectiveness of the index. Figure 6A shows an image recorded by a conventional visible camera from a cell phone camera. Figure 6B–D show processed images recorded using a SWIR hyperspectral imager in reflection mode. Low signal/noise ratio bands were removed using a spectral cropping function. A pseudo-RGB image composed from three wavelengths shows the difference between the two leaves (Figure 6B). The contrast between two leaves can be further enhanced with PCA. Three selected principal components #1/2/3 were used in a pseudo-RGB format as red, green and blue (Figure 6C). Combined in a single image, these components highlight the difference between the two leaves. Even higher contrast can be achieved using a previously developed index of drought using the formula: *I* = (1529–1416 nm)/(1519 + 1416 nm)^36^ (Figure 6D). This can be achieved by selecting Mathematics from the ANALYSIS section, then selecting Michelson Ratio and finally selecting two wavelengths 1416 nm and 1529 nm.

### Geospatial applications

**Figure 7.**
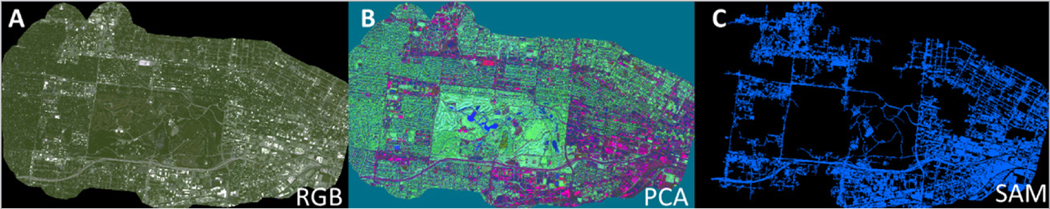
Multispectral image of the St Louis area with four bands: RGB + NIR: 430–550 nm (blue),490–610 nm (green); 600–720 nm (red); 750–950 nm (NIR). A: RGB image; B: Principal Component Analysis (PCA); C: Spectral Angular Mapping with one class selected. Satellite Sensor Pleiades-1B (0.5 m) operated by Airbus Defence & Space. The data were acquired from Apollo Hunter. Example file can be downloaded through IDCube Lite under File/Example Files/St Louis area.

Geospatial remote sensing is one of the more mature applications of hyperspectral imaging due to its relatively long history, beginning in the middle of 1970s.^37^ Since then, a large number of platforms based on satellites, planes and, recently, drones have been developed. IDCube Lite can be used on any of these platforms. The current version of the software enables data processing from hyperspectral and multispectral satellites such as ER-01 Hyperion,^38^ Sentinel-2,^39^ Orbita satellites,^40^ airborne systems such as AVIRIS^41,42^ and other platforms. The user first downloads the file from the relevant image provider websites. IDCube Lite converts the unzipped data into the IDCube format, saves and automatically opens the converted file for further processing. An example of this workflow is shown in Figure 7, where the original dataset was first downloaded from a commercial vendor (Apollo Hunter), converted to the IDCube format and processed to produce an RGB image using the first three bands (Figure 7A). The dataset was then processed by PCA. For better visualisation of the objects of interest, three principal components were used to construct a pseudo-RGB image (Figure 7B). A PCA-based datacube was further classified using a SAM method by selecting road as endmember spectra to generate an image of roads and streets (Figure 7C).

## Future development

Our current version of IDCube Lite is downloadable free software with the performance limited by the user’s computational resources. The future IDCube platform will address the need for a web-accessible platform to perform complex and computationally demanding tasks in real-time. Equipped with advanced image processing and machine learning capabilities, the web–based, constantly updated IDCube platform will enable geospatial, biomedical and other scientists and stakeholders to perform sophisticated analysis without significant computational resources, using only conventional desktops or laptops.

## Supplementary Material

Supplementary

## Figures and Tables

**Table 1.** List of functionalities offered by IDCube Lite, specific functions and usage case. Visually, these functionalities are mapped to panels shown in Figure 1.^a^

Modules	Specific operations	Usage example
Data I/O	Import, convert, export	Import satellite and other images and datasets
Visualisation	Pan, zoom	Displays image
Preprocessing	Cropping, binning	Size decrease
Data reduction	PCA, MNF	Dimensional reduction
Image enhancement	Histogram-based contrast adjustment	Enhance object contrast for visualisation
Spectral analysis	Pixel selection, Spectral Matching, endmembers etc.	Find pixels with similar spectral signatures in image
Segmentation	Thresholding	Spectral correlation and divergence maps
Image algebra	Addition, subtraction etc.	Contrast enhancement

a see Supplementary Information describing the steps

**Table 2.** Description of the datasets used as an example.

Dataset	Application	Instrument	# Channels and wavelengths	Size (MB)
Random items	Machine vision	Benchtop, SWIR/pushbroom	510 channels 867–1700 nm	326
Human hand	Biomedical	Benchtop, SWIR/pushbroom	343 channels 950–1700 nm	217
Leaves	Agriculture	Benchtop, SWIR/pushbroom	350 channels 940–1700 nm	82
View of Forest Park in St Louis	Geospatial	Satellite, Pleiades-1B/AIRBUS	4 channels 430–550 nm (blue), 490–610 nm (green), 600–720 nm (red); 750–950 nm (NIR)	46

**Table 3.** Types of files and formats supported by IDCube Lite (other sources can also be recognised).

Format	Type of detectors	Source	Implementation
JPEG, PNG	Colour RGB cameras	Many	Yes

TIFF	Multi and hyperspectral satellites	AVIRIS	Yes
		AVIRIS NG^a^	In process
		OBT satellites	Yes
		Hyperion	Yes
	Bench-type imagers	Various	Yes

hdr/raw/dat	Multi and hyperspectral satellites	Hyperion	Yes
	Bench-type HSI imagers	Specim	Yes
		Middleton	Yes
		Spectral Vision	Yes
		CytoViva	Yes
		Headwall	

JP2	Multispectral satellites	Sentinel-2	Yes
h5	Multispectral satellites	Suomi NPP	In process

a AVIRIS Next Generation data have different sets of wavelengths from the AVIRIS
