# Supplementary material for: IDCube Lite: Free Interactive Discovery Cube software for multi- and hyperspectral applications
Source: J Spectr Imaging. Author manuscript; Available in PMC 2022 Jan 1. (PMC8409277; doi:10.1255/jsi.2021.a1)
Supplement: Supplementary [file NIHMS1712850-supplement-Supplementary.pdf]

D. Mishra, H. Hurbon, J. Wang, S.T. Wang, T. Du, Q. Wu, D. Kim, S. Basir, Q. Cao, H. Zhang, K. Xu, A. Yu, Y. Zhang, Y. Huang, R. Garnett, M. Gerasimchuk-Djordjevic and M.Y. Berezin, "IDCube Lite: Free Interactive Discovery Cube software for multi and hyperspectral applications", *J. Spectral Imaging* 10, a1 (2021). https://doi.org/10.1255/jsi.2021.a1

SUPPLEMENTARY INFORMATION

IDCube Lite – A free Interactive Discovery Cube software for multi and hyperspectral applications

(Step-by-step guide through some of the typical workflows)

Deependra Mishra,[1] Helena Hurbon, [1,4] John Wang, [1,4] Steven T. Wang,[1,4] Tommy Du,[1] Qian Wu,[1] David Kim,[1] Shiva Basir, [1] Qian Cao,[1] Hairong Zhang, [1] Kathleen Xu,[1] Andy Yu[1], Yifan Zhang,[1] Yunshen Huang,[1] Roman Garrett,[2] Maria Gerasimchuk-Djordjevic[3], Mikhail Y. Berezin[1,4]*

[1]Department of Radiology, Washington University School of Medicine, 4515 McKinley Ave, St. Louis, MO 63110; [2]Department of Computer Science and Engineering, Washington University, 1 Brookings Hall, St. Louis, MO 63110; [3]Art and Design Department, Missouri State University, 901 S. National Ave, Springfield, MO 65897, [4]HSpeQ LLC, 4340 Duncan Ave, St. Louis, MO 63110
Corresponding author: berezinm@wustl.edu

Contents

1. Data Import/Reduction/Correction/Export 2
2. Visualization 3
3. Data Reduction 5
4. Image Enhancement 6
5. Spectral Analysis 7
6. Image Algebra 9
7. Spectral Signature Matching 10
8. Endmembers 12
9. Classification 14

1. Data Import/Reduction/Correction/Export

Step 1: Locate the Data section in the upper left-hand corner of the software. The section is highlighted in yellow in the image below.

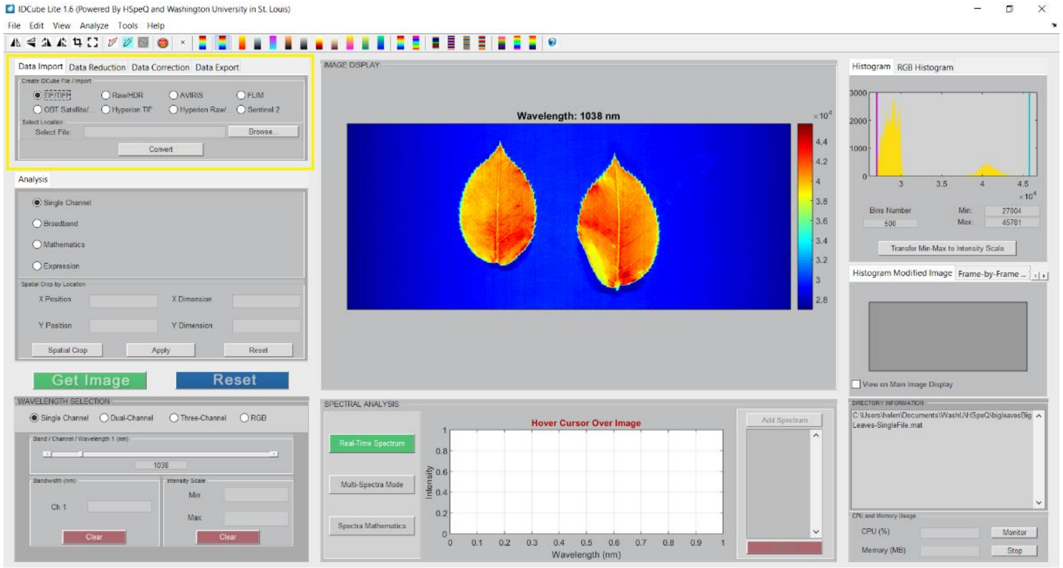

Step 2: To convert your dataset into the IDCube format, use the Data Import tab.

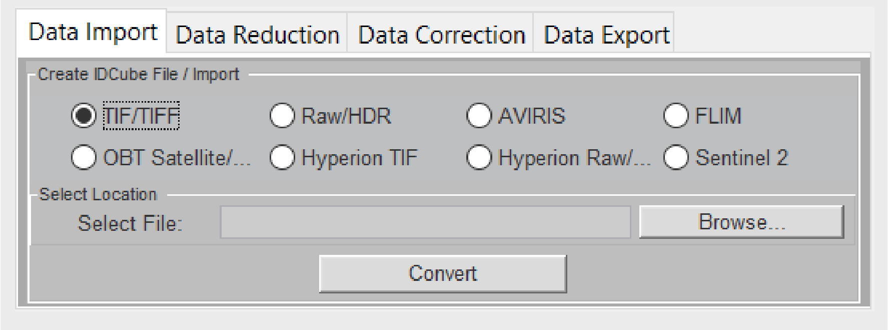

Step 3: To crop or make spatial/spectral changes to your dataset, use the Data Reduction tab.

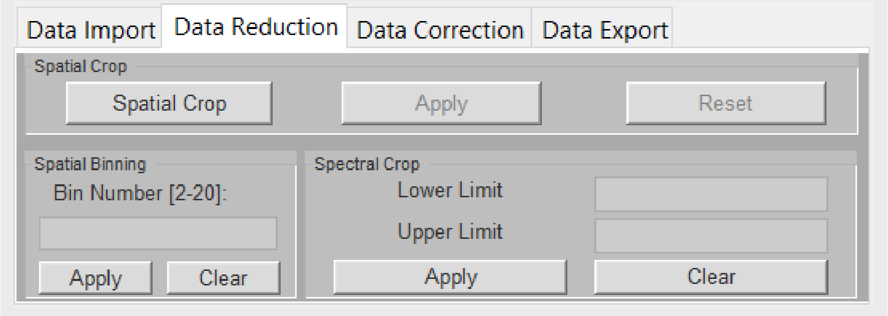

Step 4: Adjust the region of interest and click Apply. Reset to the original size. Alternatively, one can do cropping by providing coordinates using Spatial Crop by Location,

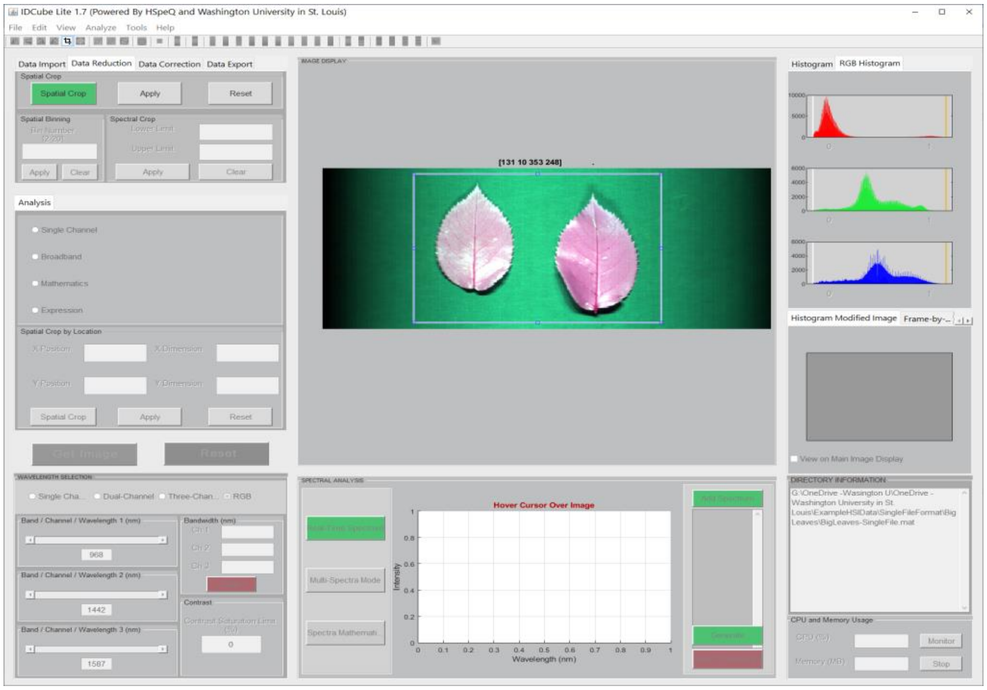

2. Visualization

Step 1: Locate the Image Display section in the middle of the software. The section is highlighted in yellow in the image below.

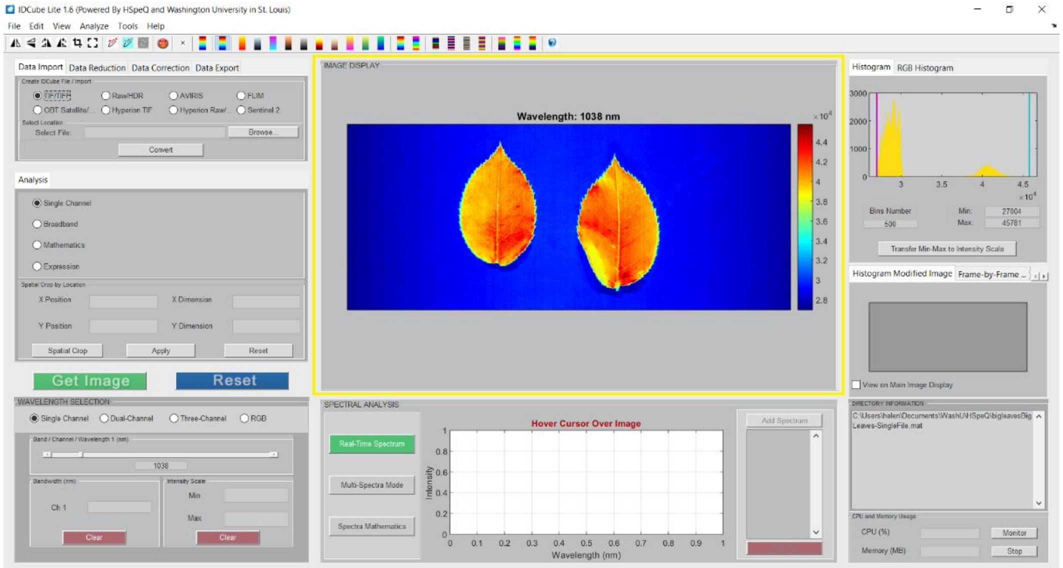

Step 2: Locate and click on the icon to show the Keyboard Shortcut for the Zoom and Pan help menu.

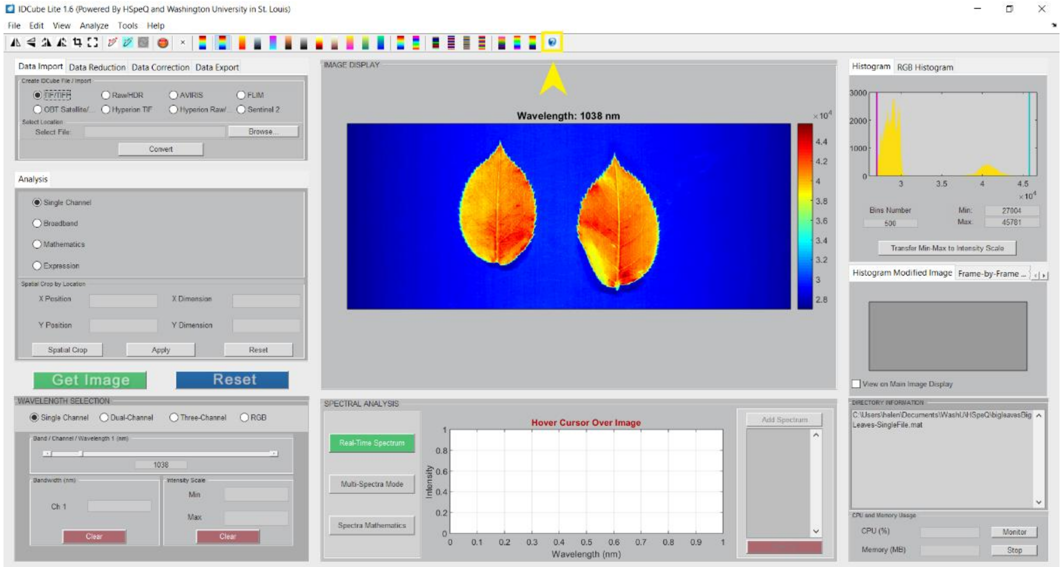

Step 3: The help menu will appear once the question mark icon is clicked.

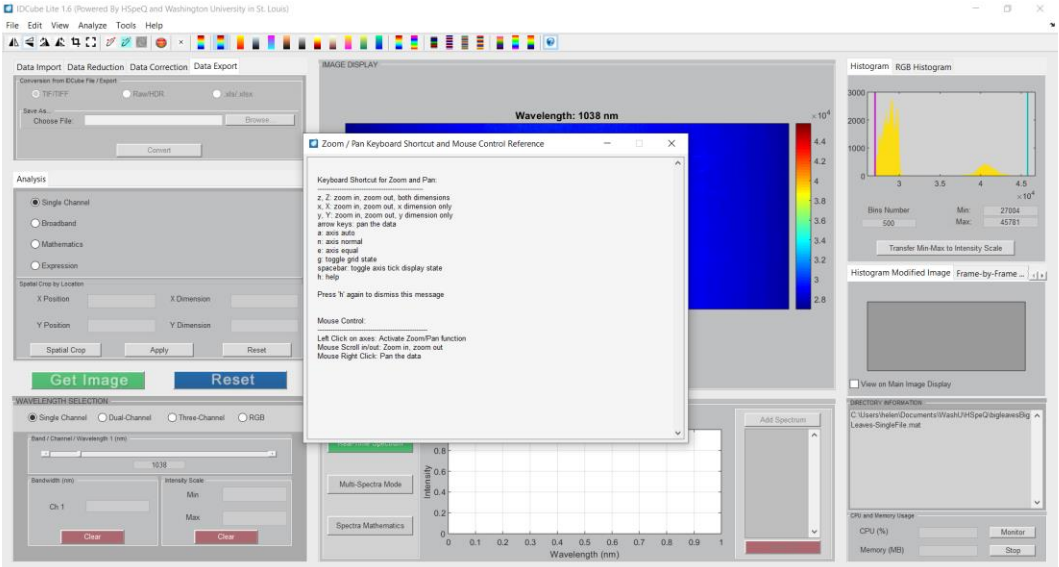

Step 4: Read the help menu to utilize the pan and zoom functions.

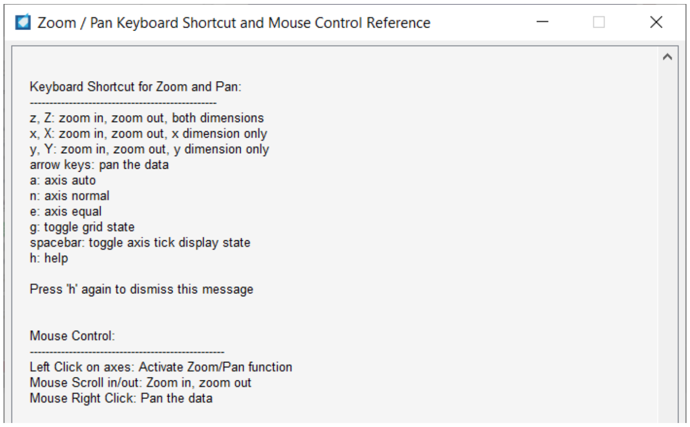

3. Data Reduction

Step 1: Hover mouse over the Analysis menu option at the top of the software. Click on Analyze.

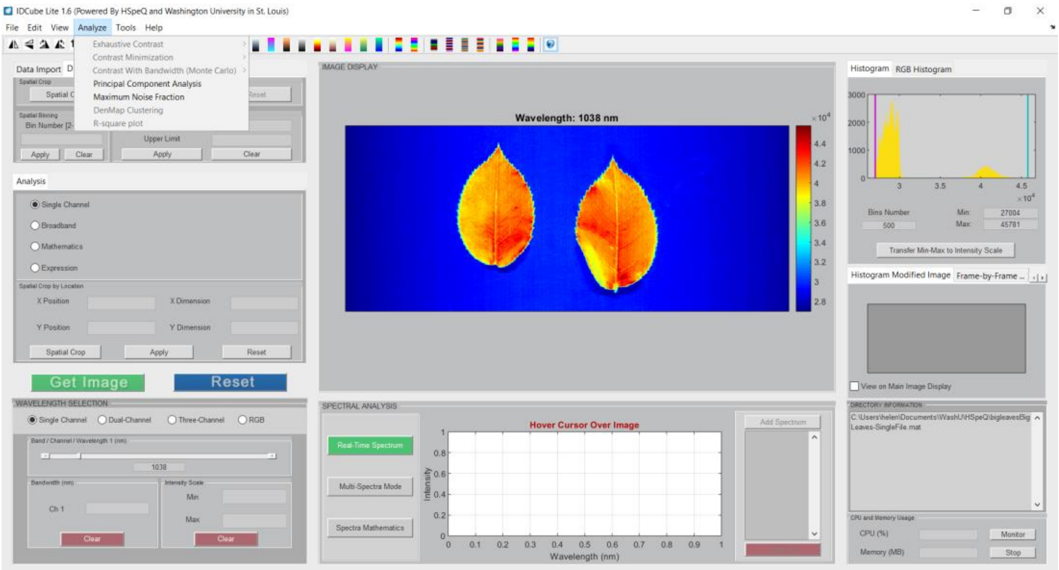

Step 2: To reduce your data using Principal Component Analysis, click on Principal Component Analysis (or Maximum Noise Fraction to also reduce noise)

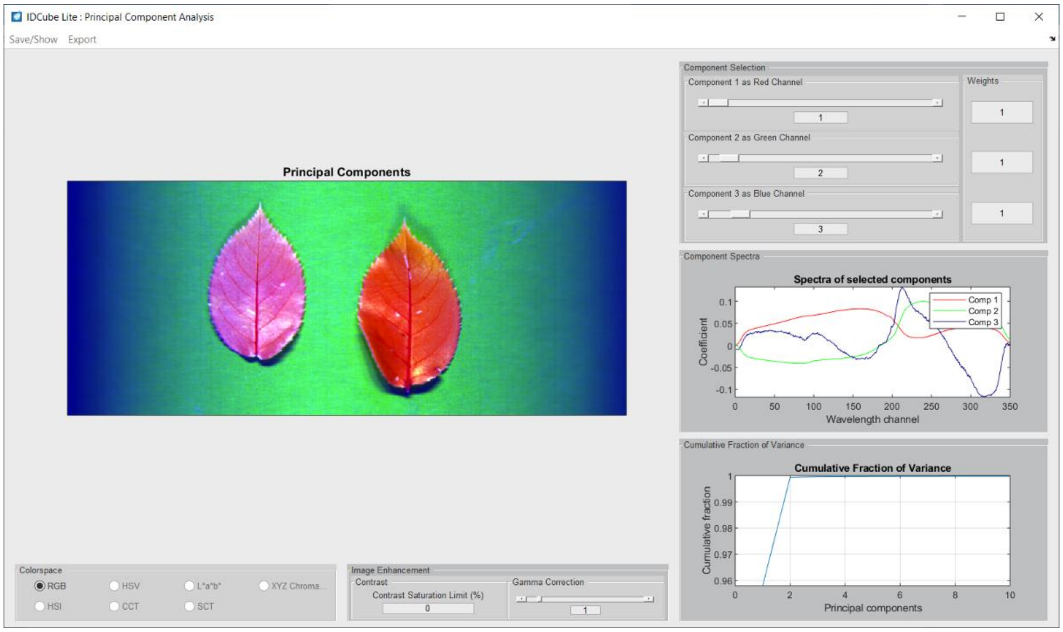

4. Image Enhancement

Step 1: To perform Image Enhancement, locate the Histogram section in the upper right-hand corner of the software. The section is highlighted in yellow in the image below.

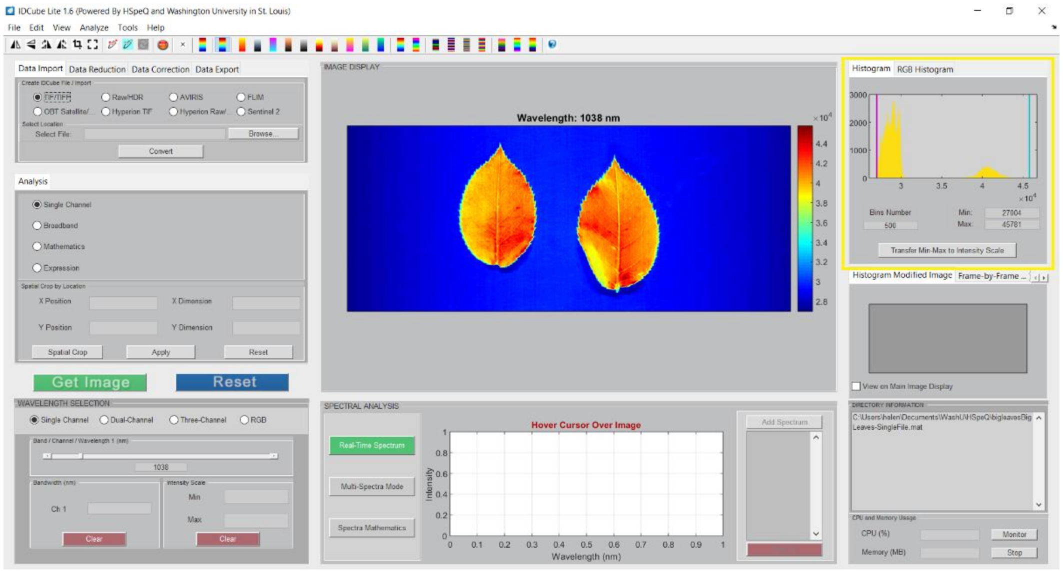

Step 2: To adjust the image contrast using the histogram, move the purple and turquoise vertical lines left and right.

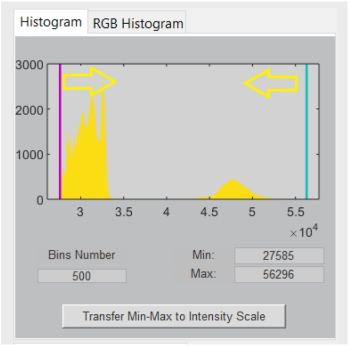

Step 3: To adjust the image contrast using the RGB histogram, move the white and yellow vertical lines left and right on each channel.

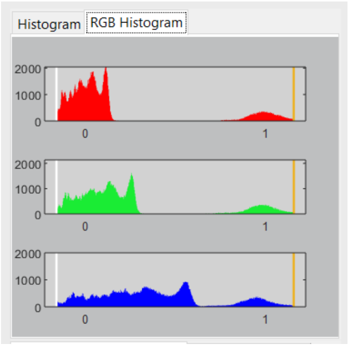

5. Spectral Analysis

Click Real time Spectra and hover over the image. Click again to freeze the changes. Click Multispectral Mode to select any number of Regions of Interests to visualize their spectra.

Step 1: To perform Spectral Analysis, click Spectra Mathematics tab on the Spectral Analysis plane. That will enable selection up to two regions of interest and visualize their spectra.

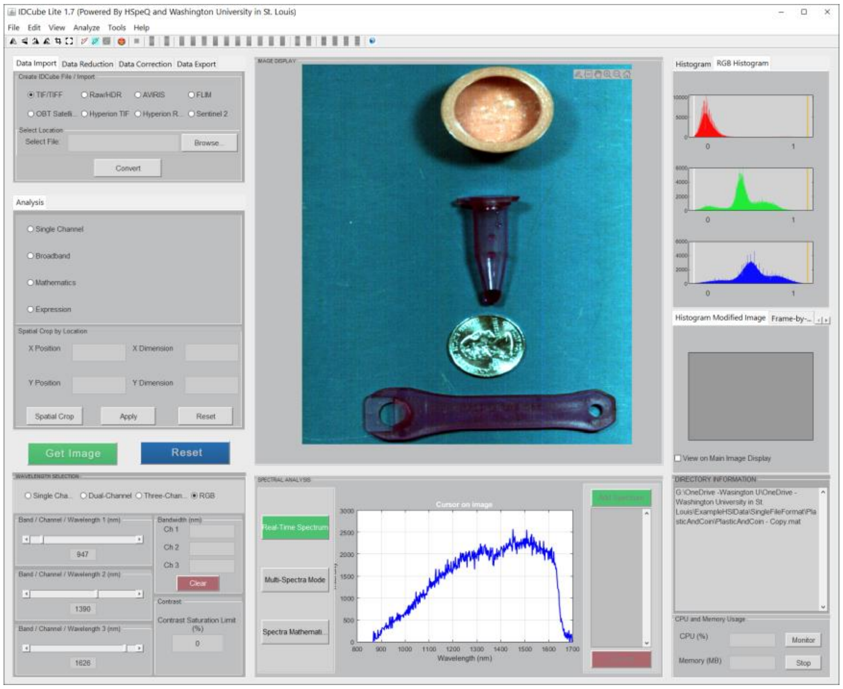

Step 2: Select the function from the menu and click Generate. Some functions require only one spectrum selected.

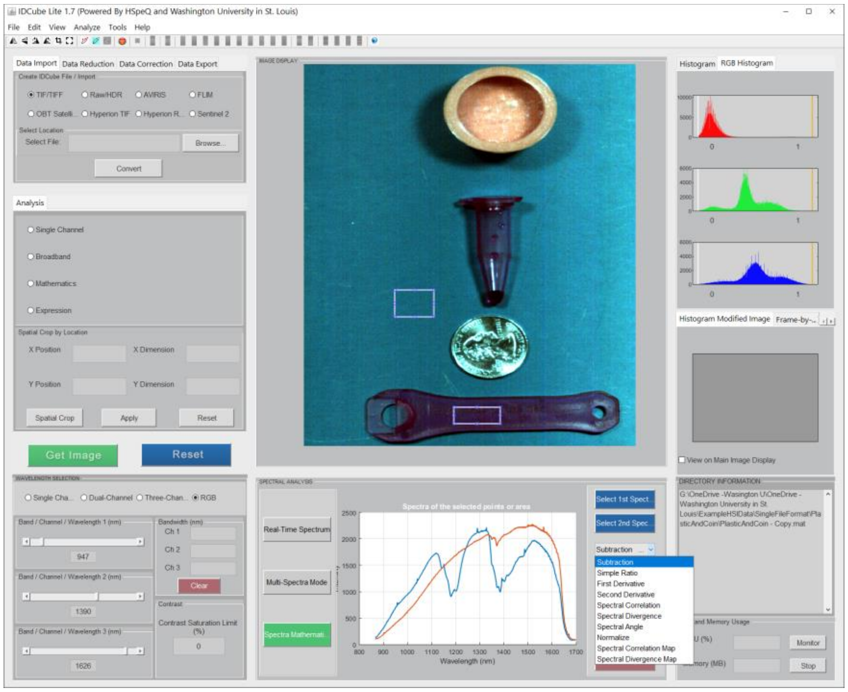

Step 3: Move a mouse over the spectra to activate a popup toolbar in the Spectral Analysis panel. To copy the spectra to the Excel, first select the brush from the toolbar in the right corner (hidden) and brush over the entire spectra. Right click and select Copy to Clipboard.

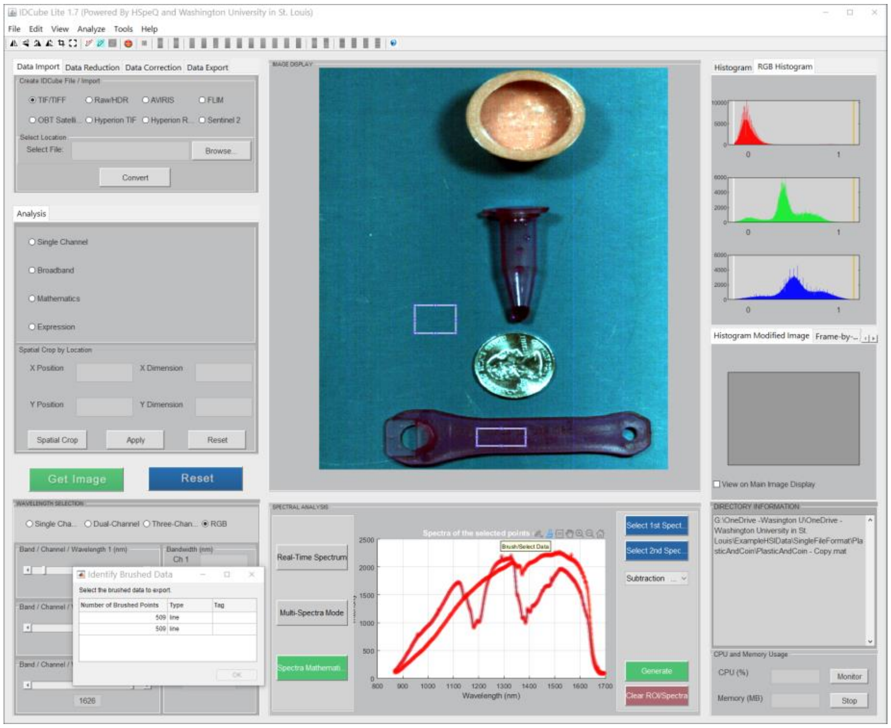

Step 4: In the popup window Identify Brushed Data select the line of interest and click OK. Open Excel or any graph analysis software and paste your data. Repeat for another line of you have more than one spectrum.

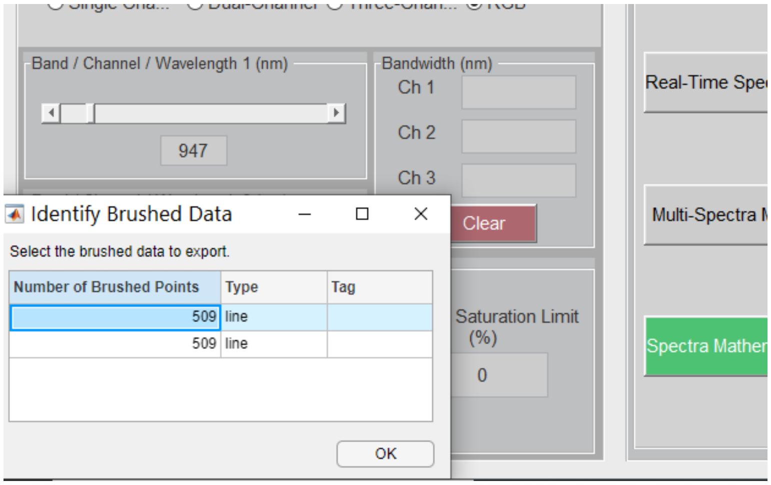

6. Image Algebra

Step 1: To perform Image Algebra, navigate to the Analysis section on left-hand side of the software. The section is highlighted in yellow in the image below.

Step 2: Click the radio button for Mathematics.

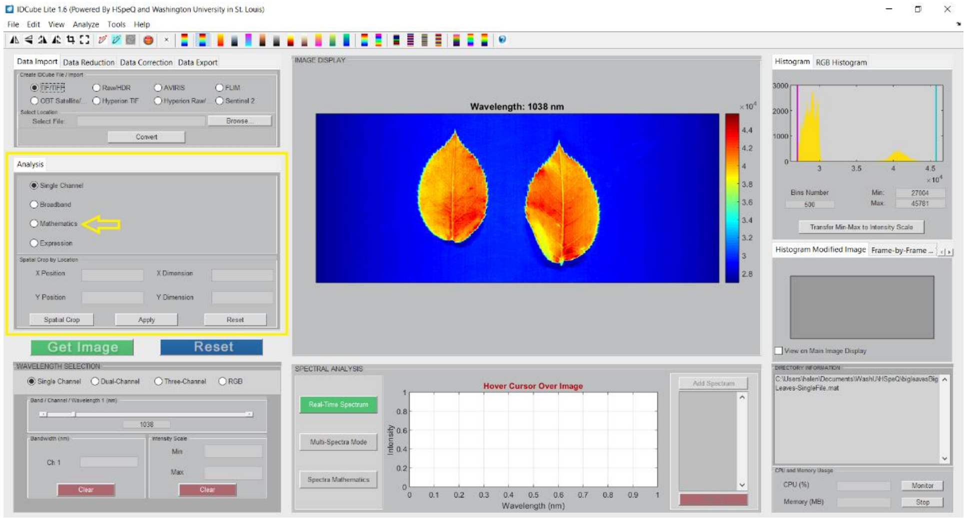

Step 3: Click on the down arrow on the right-hand side of the white bar.

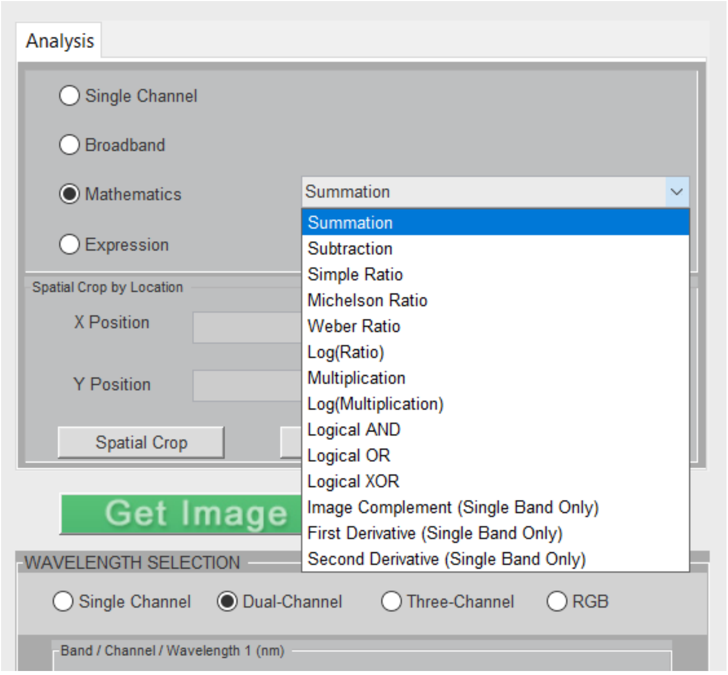

Step 4: Click on which mathematical function to perform on the dataset.

7. Spectral Signature Matching

Step 1: Open a hyperspectral imaging file through the main interface. Navigate to Tools – Spectral Signature Matching

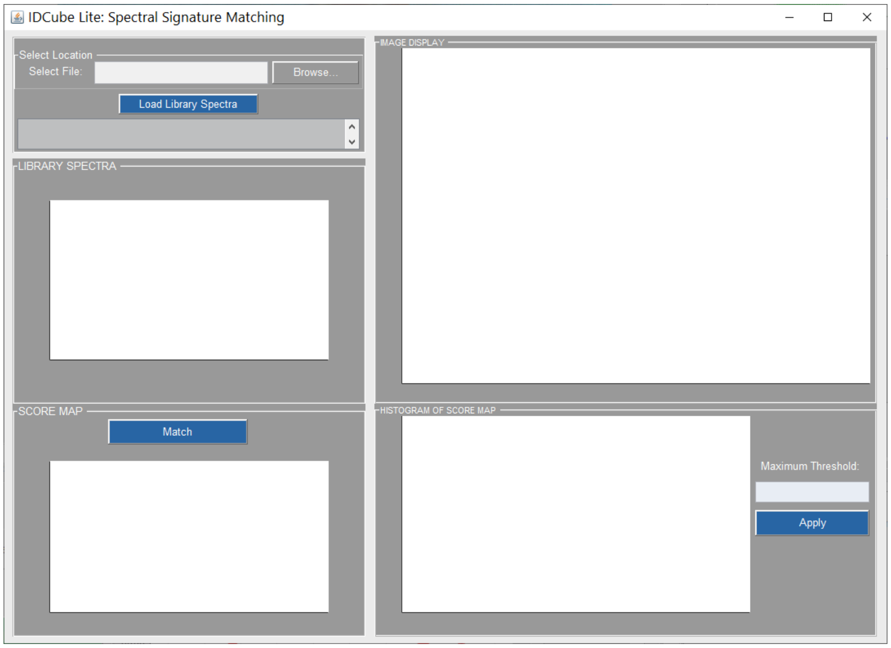

Step 2: Select a spectrum from a library or your own saved spectrum. The spectrum file should be a txt file and must be named as NameOfFile_spectrum.txt (suffix spectrum.txt is mandatory).

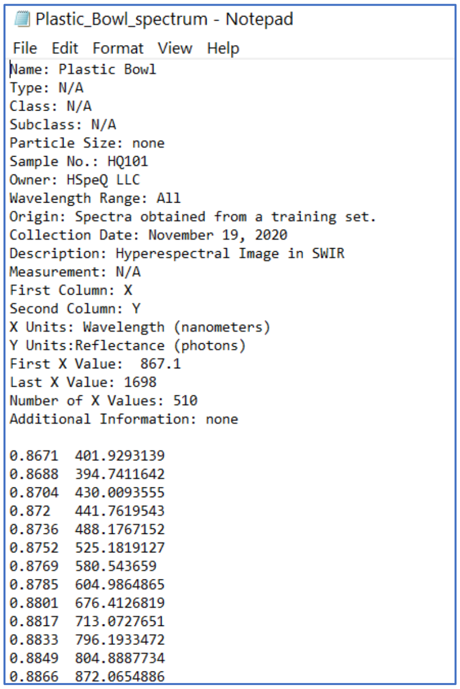


```
Name: Plastic Bowl
Type: N/A
Class: N/A
Subclass: N/A
Particle Size: none
Sample No.: HQ101
Owner: HSpeQ LLC
Wavelength Range: All
Origin: Spectra obtained from a training set.
Collection Date: November 19, 2020
Description: Hyperespectral Image in SWIR
Measurement: N/A
First Column: X
Second Column: Y
X Units: Wavelength (nanometers)
Y Units:Reflectance (photons)
First X Value:  867.1
Last X Value: 1698
Number of X Values: 510
Additional Information: none

0.8671  401.9293139
0.8688  394.7411642
0.8704  430.0093555
0.872   441.7619543
0.8736  488.1767152
0.8752  525.1819127
0.8769  580.543659
0.8785  604.9864865
0.8801  676.4126819
0.8817  713.0727651
0.8833  796.1933472
0.8849  804.8887734
0.8866  872.0654886
```

For geospatial applications, the spectrum files can be received thorough https://speclib.jpl.nasa.gov/library that features a collection of more than 3400 unique spectra. IDCube also allows to save spectra (from IDCube Spectral Analysis, or Endmembers spectra).

These spectra need to be first converted into the correct format showing above. An example of the spectra Plastic_Bowl_spectrum.txt can be downloaded from https://www.idcubes.com/examples

Step 3: Press Load Library Spectra

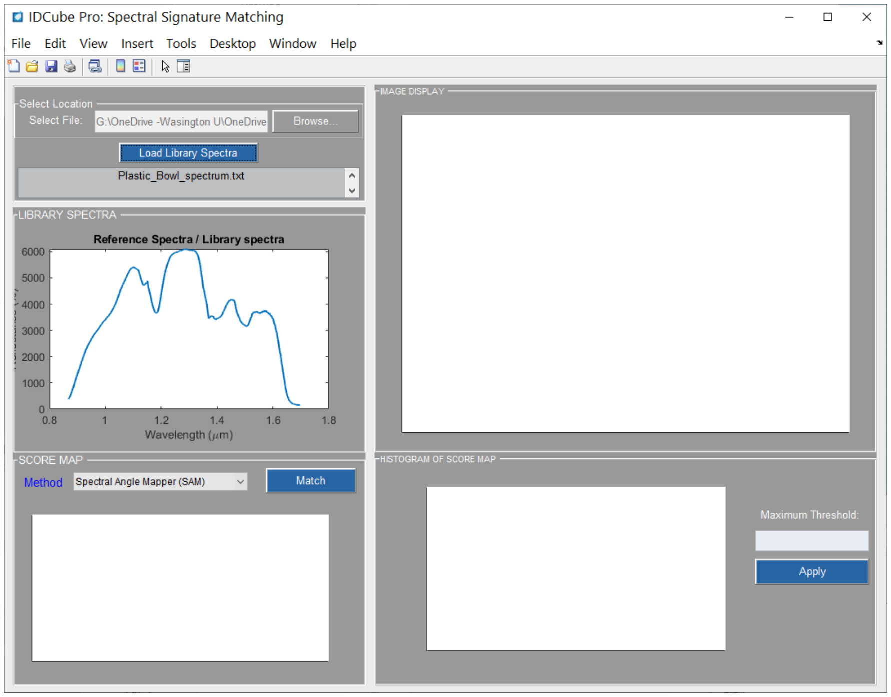

Step 4: Click Match to populate a SCORE MAP. That might take several minutes for large files. Lower score corresponds to a better match.

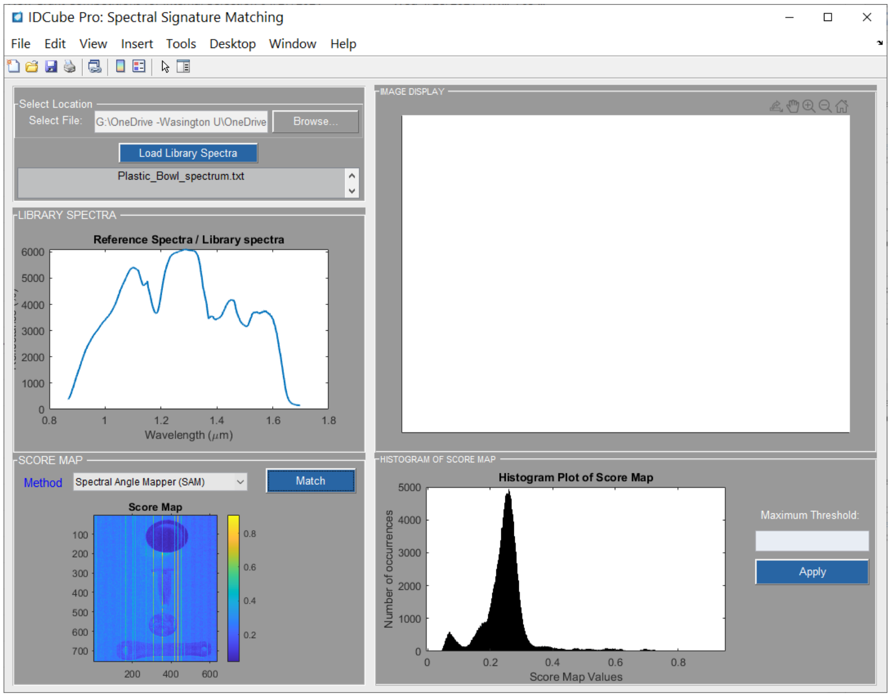

Step 5: Put a Maximum Threshold that corresponds to a specific feature at the histogram, normally in a valley. For example, 0.12 max threshold will represent all pixels with the score map values from 0 to 0.12.

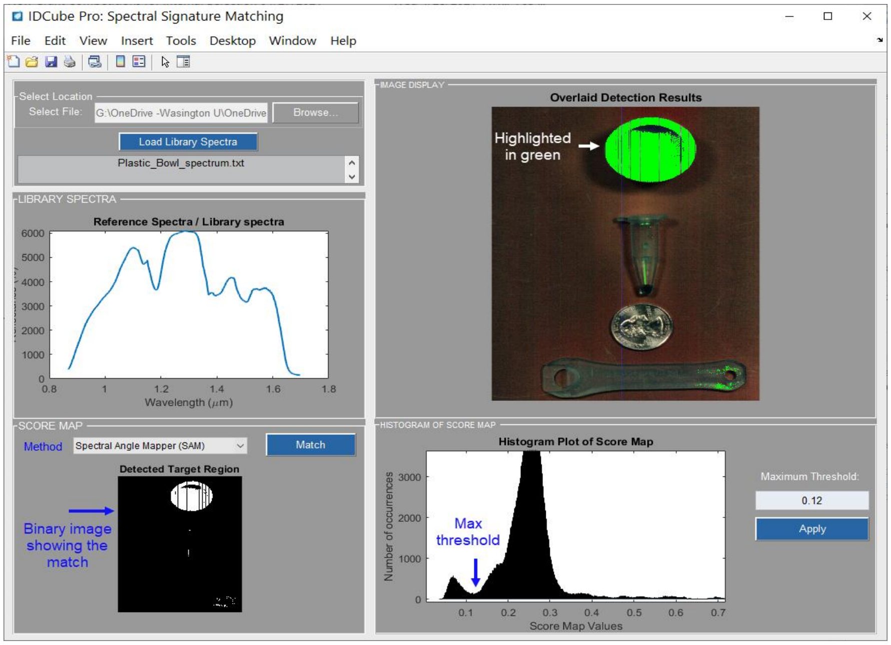

Hint: All panels are interactive. Click on the spectrum, images, or the histogram to zoom in and out (z, ctrl-z) or change the axes (x, ctrl- x;, y, ctrl-y) or move.

8. Endmembers

Step 1: Open a hyperspectral imaging file through the main interface. Navigate to Tools – Endmembers

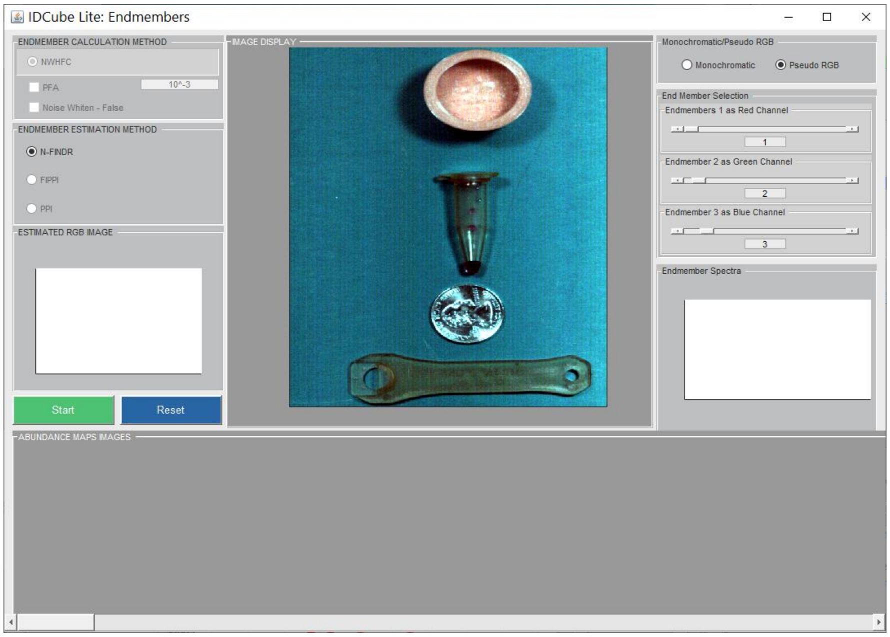

Step 2: Select a method for the Endmembers number calculation (the default for IDCube Lite is MWHFC), then selected the endmember estimation method (the default for IDCube Lite is N-FINDR) and click Start. The panels show all identified endmember images through their Abundance Maps, and individual spectral. The app also shows an image corresponding to the best combination of three endmembers (Estimated RGB Image).

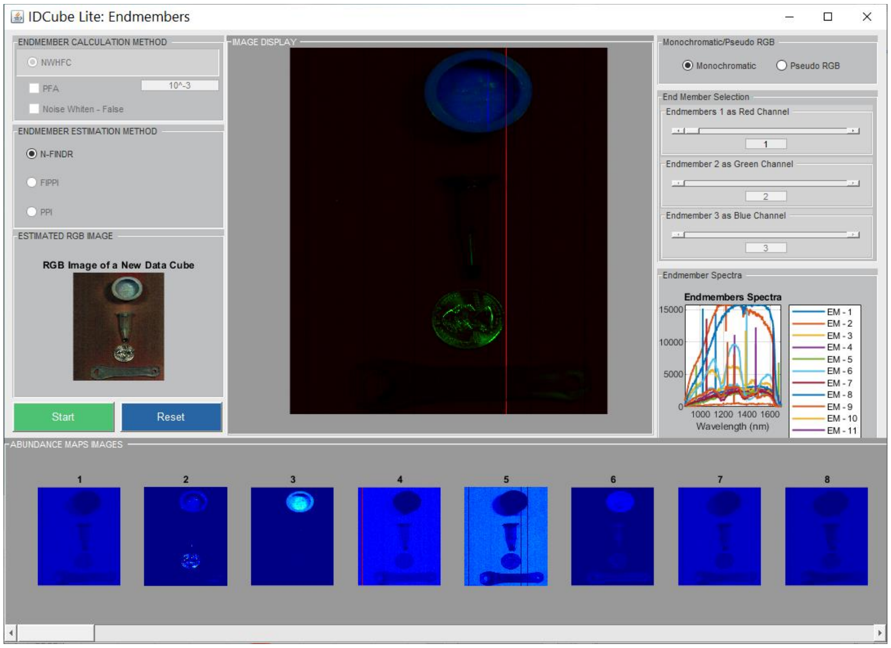

Step 3: Select Monochromatic or RGB radio buttons and scroll to visualize a single or a combination of up to three endmembers in the Image Display.

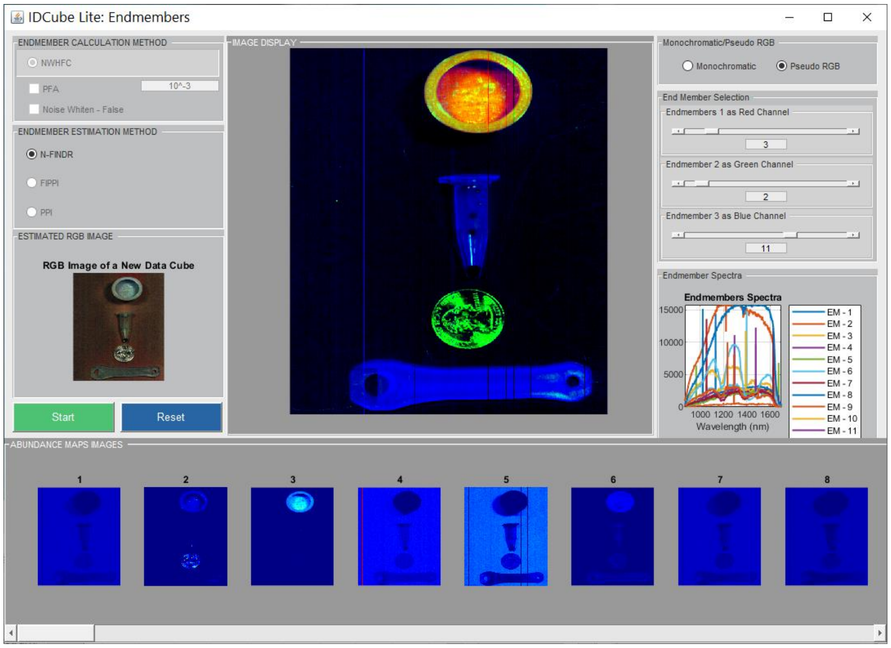

9. Classification

Step 1: Open a hyperspectral imaging file through the main interface. Navigate to Tools – Classifications. Select either a Spectral Angular Mapping or Least Square algorithms.

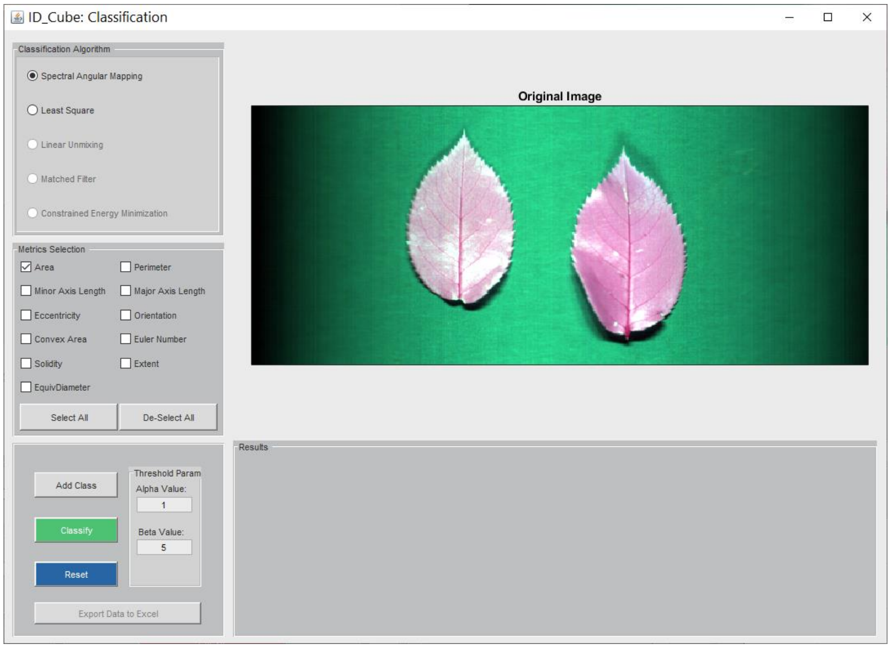

Step 2: Click Add Class and Select a Region of interest. Add Class again and select another R0I. You can select many R0Is. Click Classify to perform classification. Adjust the output by specifying Threshold Parameter Alpha Value.

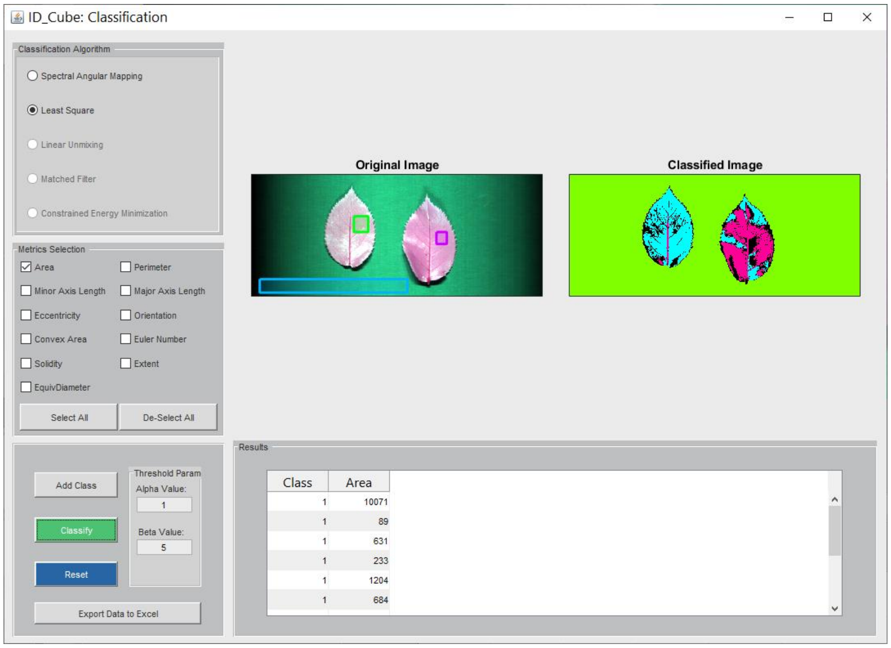

Note: the colors in the classified image are assigned randomly. To change the colors click Classify again without changing any other parameters. If you need to move the R01 or add another Class after you performed classification, please click Reset and repeat **Step 2**.

Step 3: To copy results to Excel, click Export Data to Excel. Alternatively, highlight the results with Ctrl-A and Copy. Paste the results to Excel

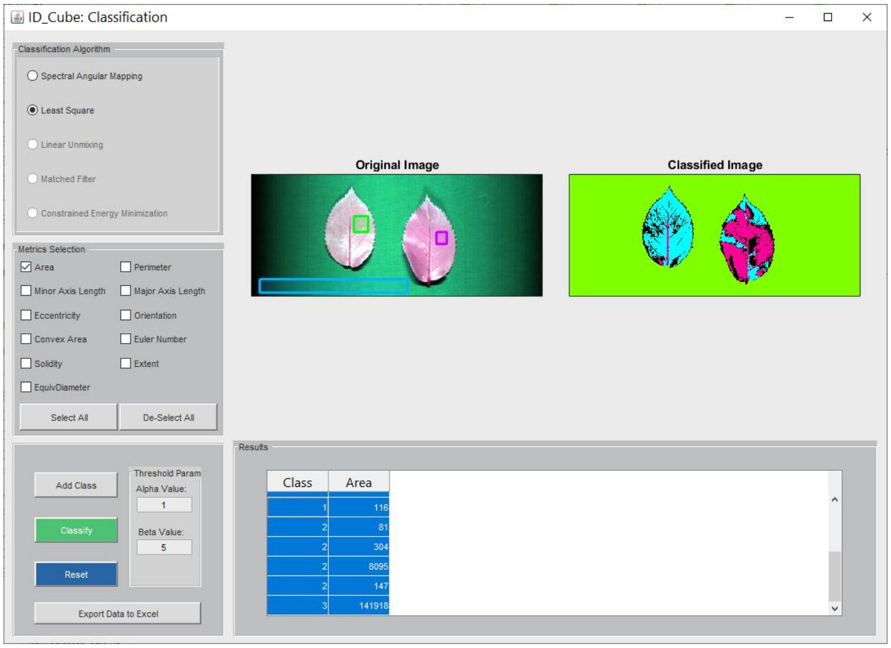

PLEASE NOTE THAT THIS IS NOT A COMPLETE GUIDE TO ALL THE FUNCTIONS THAT IDCUBE LITE OFFERS. CHANGES MIGHT BE INTRODUCED TO THE FUTURE RELEASES OF THE SOFTWARE.
